# Measuring aging rates of mice subjected to caloric restriction and genetic disruption of growth hormone signaling

**DOI:** 10.18632/aging.100919

**Published:** 2016-03-07

**Authors:** Jacob J.E. Koopman, Diana van Heemst, David van Bodegom, Michael S. Bonkowski, Liou Y. Sun, Andrzej Bartke

**Affiliations:** ^1^ Section of Gerontology and Geriatrics, Department of Internal Medicine, Leiden University Medical Center, Leiden, the Netherlands; ^2^ Leyden Academy on Vitality and Ageing, Leiden, the Netherlands; ^3^ Division of Geriatric Research, Department of Internal Medicine, Southern Illinois University School of Medicine, Springfield, IL 62794-9628, USA; ^4^ Paul F. Glenn Laboratory, Department of Biology, Massachusetts Institute of Technology, Cambridge, MA 02139, USA; ^5^ Department of Biology, University of Alabama at Birmingham, Birmingham, AL 35294, USA

**Keywords:** aging, aging rate, mice, caloric restriction, growth hormone, Gompertz model

## Abstract

Caloric restriction and genetic disruption of growth hormone signaling have been shown to counteract aging in mice. The effects of these interventions on aging are examined through age-dependent survival or through the increase in age-dependent mortality rates on a logarithmic scale fitted to the Gompertz model. However, these methods have limitations that impede a fully comprehensive disclosure of these effects. Here we examine the effects of these interventions on murine aging through the increase in age-dependent mortality rates on a linear scale without fitting them to a model like the Gompertz model. Whereas these interventions negligibly and non-consistently affected the aging rates when examined through the age-dependent mortality rates on a logarithmic scale, they caused the aging rates to increase at higher ages and to higher levels when examined through the age-dependent mortality rates on a linear scale. These results add to the debate whether these interventions postpone or slow aging and to the understanding of the mechanisms by which they affect aging. Since different methods yield different results, it is worthwhile to compare their results in future research to obtain further insights into the effects of dietary, genetic, and other interventions on the aging of mice and other species.

## INTRODUCTION

Extensive experiments have demonstrated that caloric restriction and genetic disruption of growth hormone signaling can profoundly counteract aging in mice [1]. Caloric restriction — or dietary restriction — is an environmental intervention, whereby the usual *ad libitum* dietary intake is limited to an intake of 30-40% less. Mice subjected to caloric restriction can live up to 60% longer, suffer less often and at higher ages from age-associated disorders, and exhibit less molecular stress and damage [2, 3]. Disruption of growth hormone signaling is a genetic intervention, whereby the production of growth hormone-releasing hormone, growth hormone, or the receptor of growth hormone is impaired, so that the effects of growth hormone are annulled. Mice with disrupted growth hormone signaling can live up to 70% longer, suffer less often and at higher ages from age-associated disorders, have youthful metabolic characteristics such as a higher insulin sensitivity, and have an enhanced resistance against molecular-genetic stress and damage [4-7].

A population's aging is defined as an increase in the risk of death with increasing age [8, 9]. Following this definition, the effects of caloric restriction and genetic disruption of growth hormone signaling on aging are generally examined through age-dependent survival or age-dependent mortality. Age-dependent survival can easily be determined and depicted for relatively small populations and reveals the effects on life expectancy. However, since age-dependent survival and life expectancy do not reveal at which ages and to what extent the risk of death increases, they conceal the effects on aging [10-12]. Age-dependent mortality reveals the effects on aging when it is expressed as an age-dependent mortality rate, which describes the age-dependent risk of death. Age-dependent mortality rates are generally fitted to the Gompertz model, after which they increase linearly with age on a logarithmic scale. The linear increase of such a modeled mortality rate is classically interpreted as an aging rate [8, 10]. However, the use of the Gompertz model constrains mortality rates to increase linearly on a logarithmic scale, which may not correspond with the increases in the crude age-dependent mortality rates, especially in relatively small populations [13, 14]. Moreover, it has been demonstrated theoretically and empirically that the linear increase in a mortality rate on a logarithmic scale, as modeled by the Gompertz model, is an inaccurate measure of the aging rate [15-18]. Therefore, alternative methods are needed to accurately examine the effects of interventions such as caloric restriction and genetic disruption of growth hormone signaling on age-dependent mortality rates.

We have validated a method to derive aging rates from the increase in a mortality rate with age on a linear scale instead of a logarithmic scale [16, 17]. We have substantiated elsewhere that aging is measured more accurately on a linear than a logarithmic scale [18]. This method can be applied without the need to fit the mortality rates to the Gompertz model or any similar model [14]. In this manner, the method does not assume mortality rates to conform to any specific age pattern, but is sensitive to changes in the crude mortality rates at all ages and is solely based on and closely aligns with the definition of aging as an increase in the risk of death with age. As an advantageous consequence, the method is applicable to populations of any species and of relatively small sizes. Here we compare this method with the classically used method in order to examine the effects of caloric restriction and genetic disruption of growth hormone signaling on murine aging.

## RESULTS

We derived aging rates from age-dependent mortality rates in two study populations of mice, referred to as Population A and Population B. Both populations included four groups of mice subjected to caloric restriction, disruption of growth hormone signaling, both, or none of these interventions. In Population A, caloric restriction entailed an intake of 30% less than the *ad libitum* dietary intake and growth hormone signaling was disrupted by knockout of the growth hormone receptor gene *Ghr/Ghrbp* [19]. In Population B, caloric restriction entailed an intake of 40% less than the *ad libitum* dietary intake and growth hormone signaling was disrupted by knockout of the growth hormone-releasing hormone gene *Ghrh* [20]. The sizes and life expectancies of the four groups of mice in each population are given in Table 1.

**Table 1:** Life expectancies of mice subjected to caloric restriction and/or genetic disruption of growth hormone signaling

**Caloric restriction**	−	+	−	+
**Disruption of growth hormone signaling**	−	−	+	+
**Population A**				
Number of mice	41	43	38	39
Median life expectancy	903	1127	1181	1188
Increase in median life expectancy	Ref.	25%	31%	32%
Maximal life expectancy	1275	1395	1379	1462
Increase in maximal life expectancy	Ref.	9%	8%	15%
**Population B**				
Number of mice	108	105	97	102
Median life expectancy	634	794	931	1056
Increase in median life expectancy	Ref.	25%	47%	67%
Maximal life expectancy	1171	1307	1308	1537
Increase in maximal life expectancy	Ref.	12%	12%	31%

Median and maximal life expectancies are given in days. Increases in median and maximal life expectancies are given relative to the mice not subjected to caloric restriction or disruption of growth hormone signaling (Ref.).

**Figure 1:**
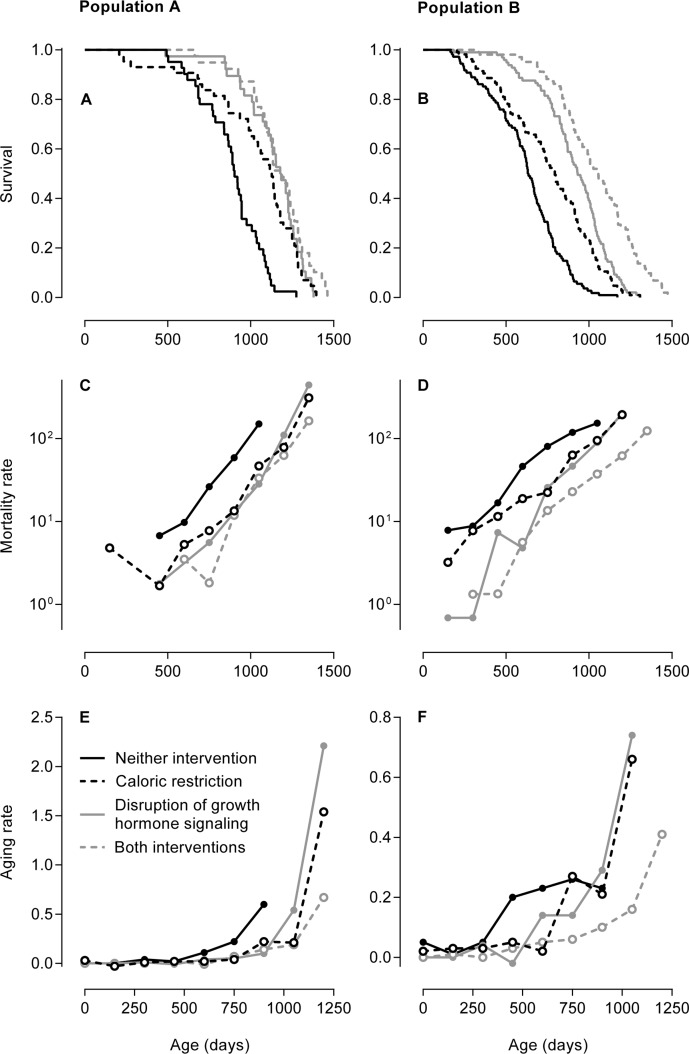
Three methods to examine the effects of caloric restriction and/or genetic disruption of growth hormone signaling on aging in mice In Population A, caloric restriction entailed an intake of 30% less than the *ad libitum* dietary intake and growth hormone signaling was disrupted by knockout of the growth hormone receptor gene *Ghr/Ghrbp* [19]. In Population B, caloric restriction entailed an intake of 40% less than the *ad libitum* dietary intake and growth hormone signaling was disrupted by knockout of growth hormone-releasing hormone gene *Ghrh* [20]. **(A, B)** Age-dependent survival of the groups of mice in Population A (**A**) and Population B (**B**) depicted as Kaplan-Meier curves. These two figures have been published previously [19, 20] and are given here as references. The copyright of Figure 1A is with the National Academy of Sciences of the USA; the copyright of Figure 1B is with the authors of the original publication [20]. **(C, D)** Age-dependent mortality rates of the groups of mice in Population A (**C**) and Population B (**D**) given on a logarithmic scale. The mortality rates are expressed in deaths per 10,000 mice per day. The linear increase in mortality rate on a logarithmic scale is classically interpreted as an aging rate. **(E, F)** Age-dependent aging rates of the groups of mice in Population A (**E**) and Population B (**F**). Contrary to the aging rates estimated by the classical method in Table 2, these aging rates were calculated without modeling the age-dependent mortality rates, describe the increases in the mortality rates on a linear scale, and are dependent on age themselves. The aging rates are expressed in deaths per 10,000 mice per day per day, which equals the change in mortality rate per day.

Figures 1A and 1B show the age-dependent survival of the groups of mice in both study populations. These two figures have been published previously [19, 20] and are given here as references. In Population A, the mice subjected to caloric restriction, disruption of growth hormone signaling, or both survived longer than the mice not subjected to these interventions. In Population B, the mice subjected to both caloric restriction and disruption of growth hormone signaling survived longest, the mice subjected to either of these interventions had intermediate survival, and the mice not subjected to these interventions survived shortest. Aging rates cannot be discerned from these figures.

Figures 1C and 1D show the age-dependent mortality rates of the groups of mice in both study populations on a logarithmic scale. In Population A, the mice subjected to caloric restriction, disruption of growth hormone signaling, or both had similar mortality rates that were lower at all ages than that of the mice not subjected to these interventions. In Population B, the mice subjected to both caloric restriction and disruption of growth hormone signaling had the lowest mortality rate, the mice subjected to either of these interventions had intermediate mortality rates, and the mice not subjected to these interventions had the highest mortality rate. The mortality rates of all groups of mice in both populations increased in a linear manner.

The linear increase in mortality rate with age on a logarithmic scale is classically interpreted as an aging rate. This linear increase is most accurately — and therefore most often — measured using the Gompertz model or a similar model [21]. We measured the linear increase in the mortality rate of each group of mice in both populations by fitting each age-dependent mortality rate to the Gompertz model. The aging rates estimated by this classical method did not differ consistently between the groups of mice in both study populations, as reported in Table 2.

**Table 2:** Aging rates of mice subjected to caloric restriction and/or genetic disruption of growth hormone signaling according to the classical method

**Caloric restriction**	−	+	−	+
**Disruption of growth hormone signaling**	−	−	+	+
**Population A**	6.1 (4.6 to 7.5)	4.9 (3.6 to 6.2)	7.6 (5.6 to 9.6)	6.4 (4.9 to 8.0)
**Population B**	4.5 (3.8 to 5.3)	3.9 (3.2 to 4.6)	5.7 (4.8 to 6.6)	4.5 (3.8 to 5.2)

Aging rates estimated by the classical method are given with 95% confidence intervals and are shown × 1000 for legibility. These aging rates are expressed in units per day. These aging rates were estimated using the Gompertz model, equal the Gompertz model's parameter γ, and describe the linear increases in the mortality rates on a logarithmic scale.

Alternatively to the aging rates estimated by the classi-cal method, we calculated aging rates as the increases in the mortality rates with age on a linear scale without fitting the mortality rates to the Gompertz model or any similar model. Figures 1E and 1F show these aging rates for the different groups of mice in both study populations. The aging rates of all groups of mice in both populations increased with age in an exponential manner. In Population A, the aging rates of the mice subjected to caloric restriction, disruption of growth hormone signaling, or both increased at higher ages and to higher levels than that of the mice not subjected to these interventions. In Population B, the aging rate of the mice subjected to both caloric restriction and disruption of growth hormone signaling increased at the highest ages to an intermediate level, the aging rates of mice subjected to either of these intervention were similar and increased at intermediate ages to the highest levels, and the aging rate of mice not subjected to these interventions increased at the lowest ages to the lowest level.

We repeated our analyses after stratifying male and female mice, which yielded similar results (data not shown).

## DISCUSSION

This study aims to examine the effects of caloric restriction and genetic disruption of growth hormone signaling on murine aging by comparing a method that calculates an aging rate as the increase in a mortality rate with age on a linear scale without the need to fit the mortality rate to the Gompertz model or any similar model with the classically used model that estimates an aging rate as the increase in a mortality rate with age on a logarithmic scale modeled with the Gompertz model. It shows that the methods yield different results. According to the classical method, these interventions negligibly and non-consistently affected the aging rates (Table 2). By contrast, according to the alternative method studied here, the aging rates of mice subjected to caloric restriction or disruption of growth hormone signaling increased at higher ages and to higher levels as compared with mice not subjected to these interventions (Figures 1E and 1F).

A key question in research on aging is whether increases in life expectancy reflect a postponement or a slowing of aging. The answer to this question is pivotal to gain insight in the mechanisms of aging, to identify interventions that modulate these mechanisms, and to predict the effects of such interventions on aging [22]. However, with respect to caloric restriction and disruption of growth hormone signaling, a clear answer to this question is lacking. While caloric restriction has long been assumed to slow aging, it is debated whether it postpones aging instead [3, 9, 23, 24]. Likewise, some presume that genetic disruption of growth hormone signaling slows aging [4], whereas others pose that it rather postpones aging [10].

Research on the effects of caloric restriction and disruption of growth hormone signaling — and other interventions — on aging is hampered by the variety of methods that measure aging through the increase in the risk of death with age [8, 9, 15, 16, 18]. The examination of age-dependent survival is unsuitable to reveal the effects of the interventions on aging and to distinguish between postponed and slowed aging [9-11, 23]. The classically used method, which fits age-dependent mortality rates to the Gompertz model and interprets their linear increases on a logarithmic scale as aging rates, suits this purpose. It identifies a slowing of aging as a flattening of a mortality rate's linear increase and identifies a postponement of aging as a lowering of a mortality rate while its linear increase remains unaltered [8, 10, 24]. However, the validity of the classical method has been disputed [15-17].

According to the classical method, caloric restriction and disruption of growth hormone signaling did not affect the aging rates, but would be interpreted to postpone aging. The alternative method studied here, by contrast, yields age-dependent aging rates that allow for a more variegated assessment beyond the question of a mere postponement or slowing of aging. This method interprets these interventions to affect the aging rates in an age-dependent manner: aging was slowed at lower ages, postponed until higher ages, but quickened at higher ages. Such a pattern resembles a compression of aging, whereby aging is postponed as well as intensified, reflected by a risk of death that increases sharply at a high age [25]. A compression of aging also becomes apparent from the life expectancies of the mice (Table 1): these interventions bring about an increase in median life expectancy that is two to four times larger than the increase in maximal life expectancy, indicating a sharper increase in the risk of death at a higher age. This effect was shared by caloric restriction and genetic disruption of growth hormone signaling. When both interventions were applied jointly, their effects were mutually reinforced only in Population B — subjected to a caloric restriction of 40% of the *ad libitum* dietary intake and knockout of the growth hormone-releasing hormone gene — but not in Population A — subjected to a caloric restriction of 30% of the *ad libitum* dietary intake and knockout of the growth hormone receptor gene. These results are in line with previous observations in these populations of mice (Figures 1A and 1B) [19, 20].

The classical method has been employed intensively to study the effects of caloric restriction on murine aging. It has yielded inconsistent results in individual studies [3, 9, 23, 26]. When these individual studies are compiled, it has yielded the conclusions that caloric restriction both postpones and slows [27] or only slows aging in mice [21], while disagreement exists over the correct application of the classical method [21]. Since the alternative method studied here yields different conclusions about the effects of these interventions on murine aging, it calls for a reevaluation of the previous studies with the use of this method.

The effects of caloric restriction and genetic disruption of growth hormone signaling on murine aging are not universally equal. They differ, for example, between sexes and genetic backgrounds of mice [5, 6, 26, 28, 29]. We found similar effects in male and female mice in two genetically distinct populations. More research is warranted to establish whether the alternative method studied here finds similar effects in other study populations of mice or, if different effects are found, which of the populations' characteristics explain the differences in the results. Moreover, the method explored here can be used to examine the effects on aging of other interventions and in other species.

This study explores the different methods to examine the effects of caloric restriction and genetic disruption of growth hormone signaling on murine aging. It is beyond the aim of this study to discuss the mechanisms through which these interventions influence aging; these mechanisms are elaborately discussed in other publications, such as those referenced here. Still, the alternative method studied here may provide insight into these mechanisms. The question whether caloric restriction and disruption of growth hormone signaling postpone or slow aging is often related to the accumulation of physical damage that is thought to underlie aging, for example due to oxidative stress. While a postponement of aging is regarded to reflect an improvement of health at all ages, only a slowing of aging is regarded to reflect a decreased pace at which damage accumulates or an increased ability with which the damage can be repaired [3, 9, 23, 24]. In addition, the age at which mortality rates start to increase is related to the pace at which damage accumulates [30]. As the alternative method points to a compression of aging, it suggests that the accumulation of damage is modulated differently at different ages and suggests that aging is modulated through more complex mechanisms than a simple increase or decrease of the pace at which damage accumulates.

In conclusion, this study shows that, depending on the method that is used, different effects are found of caloric restriction and genetic disruption of growth hormone signaling on murine aging. Whereas these interventions negligibly and non-consistently affect aging rates according to the classical method to calculate aging rates, they slow aging at lower ages and quicken aging at higher ages according to the alternative method that has been validated previously. This conclusion warrants a reevaluation of previous studies on the effects of these interventions on murine aging with the use of the alternative method. Moreover, the alternative method can be applied in future research to obtain further insights into the effects of dietary, genetic, and other interventions on aging of mice and other species.

## METHODS

### Mice

Data on the mortality of mice subjected to caloric restriction and with genetic disruption of growth hormone signaling were derived from two previous studies. Mice without a receptor of growth hormone and growth-hormone binding protein were developed by targeted disruption of the *Ghr/Ghrbp* gene in 129/Ola mice and provided by Dr. Kopchick at Ohio University [31]. These mutant mice do not express the receptor of growth hormone and are consequently resistant to growth hormone. Their phenotypically normal siblings served as controls. Both the mutant and wild-type mice were divided in two groups, of which one was fed *ad libitum* and the other was subjected to caloric restriction from 56 days of age onward. Caloric restriction was gradually introduced with an intake of 10% less than the *ad libitum* dietary intake in the initial week, 20% less in the second week, and 30% less throughout the subsequent weeks [19]. We refer to this population of mice as Population A.

Mice without production of growth hormone-releasing hormone were developed by targeted disruption of the *Ghrh* gene in mice with a mixed C57BL/6 and 129/Sv background and provided by Dr. Salvatori at Johns Hopkins University School of Medicine [32]. These mutant mice do not secrete growth hormone-releasing hormone and consequently do not secrete growth hormone. Their phenotypically normal siblings served as controls. Both the mutant and wild-type mice were divided in two groups, of which one was fed *ad libitum* and the other was subjected to caloric restriction from 84 days of age onward with an intake of 40% less than the *ad libitum* dietary intake [20]. We refer to this population of mice as Population B.

All mice were bred and housed at Southern Illinois University School of Medicine under controlled temperature (20-23°C) and light conditions (12-hours light/12-hours dark cycles) and were fed Lab Diet Formula 5001 (Nestlé Purina, St. Louis, MO). Regular testing for bacterial and viral infections was negative. Few mice appearing near death, having a tumor that bled, or having a tumor that approached 10% of the body weight were euthanized. All animal protocols were in strict accordance with the recommendations in the Guide for the Care and Use of Laboratory Animals of the National Institutes of Health and were approved by the Animal Care and Use Committee of Southern Illinois University School of Medicine.

### Mortality rates

Mortality rates were calculated per group of mice per interval of 150 days of age. Mortality rates were calculated by dividing the number of mice that died by the number of days lived by all mice in the age interval of interest. If only one mouse died in the last age interval, the corresponding mortality rate was excluded.

The age-dependent mortality rate of each group of mice was fitted to the Gompertz model using Stata/SE 12.1 (StataCorp, College Station, TX) in order to obtain estimates of the Gompertz model's parameters. The Gompertz model describes mortality rate *m* at age *t* by *m*(*t*) = α e^γ*t*^, where α and γ are the model's parameters. The linear increase in a mortality rate with age on a logarithmic scale is described by γ and classically interpreted as an aging rate.

### Aging rates

Alternatively to the classically estimated aging rates, aging rates were derived from age-dependent mortality rates per group of mice per interval of 150 days of age without fitting the mortality rates to the Gompertz model or any similar model. The aging rate in each age interval was calculated as the absolute difference in mortality rate between the age interval of interest and the subsequent age interval divided by the difference in age between both age intervals, as described previously in more detail [14].
